# Supplementary material for: Robust statistical assessment of Oncogenotype to Organotropism translation in xenografted zebrafish
Source: bioRxiv. 2025 Jun 1:2025.05.28.656734. Preprint. [Version 1] doi: 10.1101/2025.05.28.656734 (PMC12154647; doi:10.1101/2025.05.28.656734)
Supplement: 1 [file NIHPP2025.05.28.656734V1-supplement-1.pdf]

bioRxiv preprint doi: https://doi.org/10.1101/2025.05.28.656734; this version posted June 1, 2025. The copyright holder for this preprint (which was not certified by peer review) is the author/funder, who has granted bioRxiv a license to display the preprint in perpetuity. It is made available under aCC-BY 4.0 International license.

Supplementary Figures

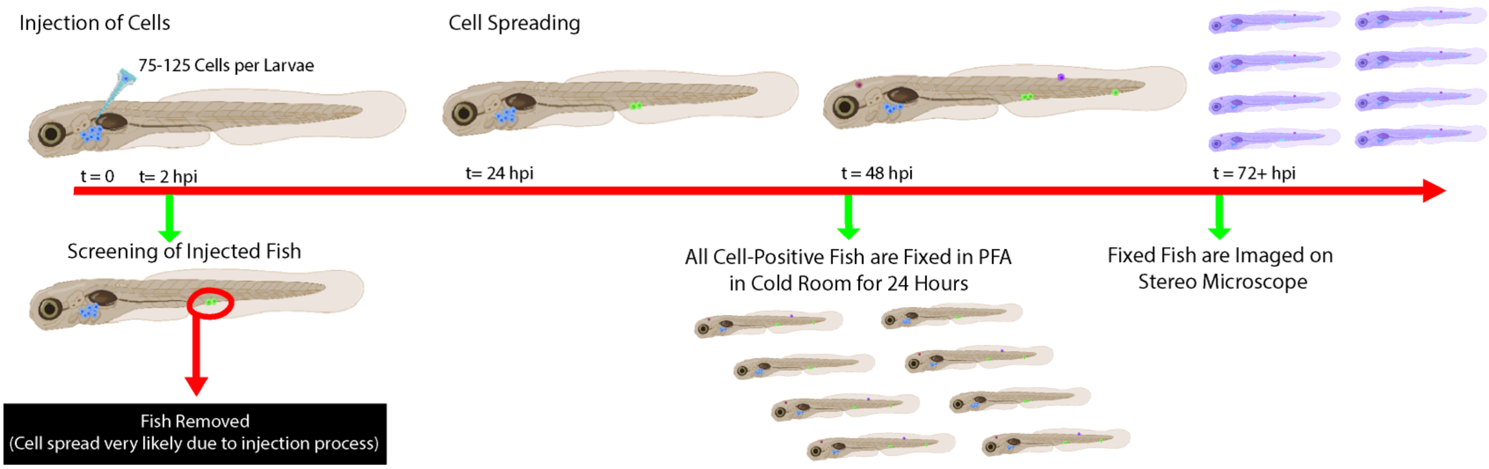

Figure S1. Cell injection workflow and processing timeline. Cells are injected at a concentration of 75-125 cells in 2-day old zebrafish larvae. Injected fish are visualized on a fluorescent stereoscope at 2 hours post injection (hpi). Fish containing cells in caudal vasculature are removed from the experimental population due to the high likelihood of cell deposition in these sites being non-biological and due to the injection process. Fish water changes (E3 media) are done every 18-24 hours along with removal of any dead larvae. Cell positive fish at 48 hpi are fixed in 4% PFA at 4°C overnight, washed and stored in PBS for imaging in fluorescent stereoscope or stored temporarily at 4°C.

bioRxiv preprint doi: https://doi.org/10.1101/2025.05.28.656734; this version posted June 1, 2025. The copyright holder for this preprint (which was not certified by peer review) is the author/funder, who has granted bioRxiv a license to display the preprint in perpetuity. It is made available under aCC-BY 4.0 International license.

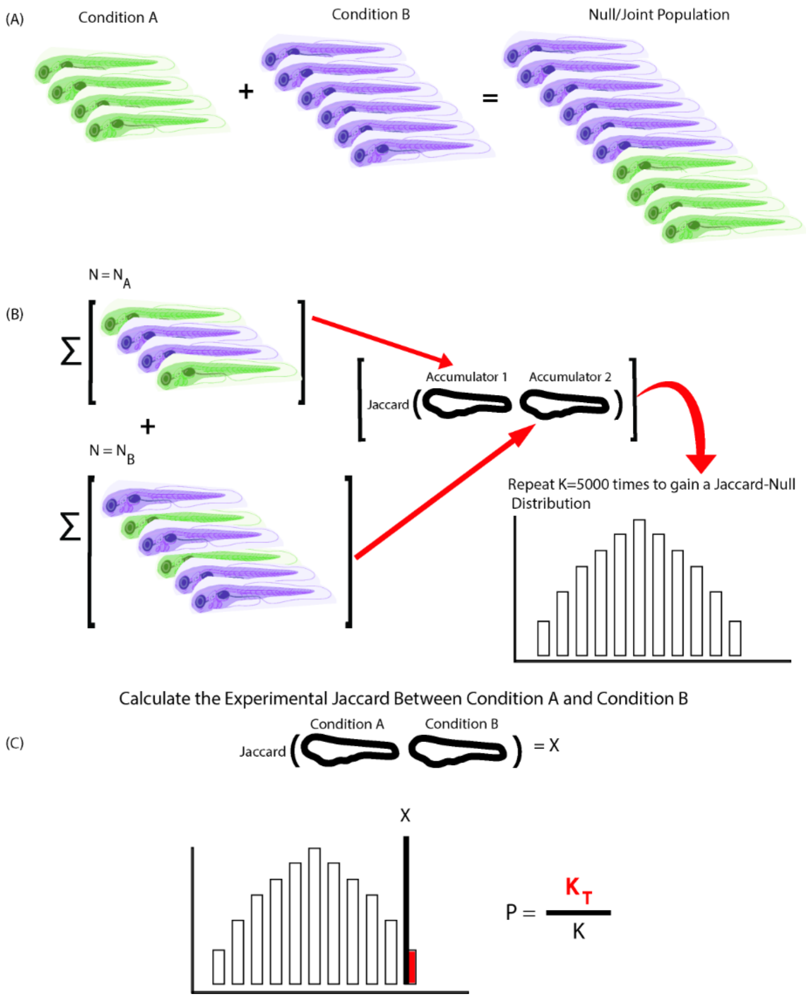

Figure S2 Statistical comparison of two Fish Metastatic Atlas pages. (A) Two fish cohorts of different experimental conditions A and B with a different number of fish (N_A,N_B) are mixed together to form a Null/joint population of N_A+N_B fish. **(B)** N= N_A and N= N_B fish are randomly sampled (with no replacement) from the joint population in (A), followed by processing of two accumulators that are compared using the Jaccard similarity coefficient. The random sampling and comparison were repeated K=5000 times to generate a Jaccard Null-distribution for the joint data set. **(C)** The experimental Jaccard value X is computed between Condition A and Condition B accumulators. P-value is defined as the number of events in the Null-distribution less than X (K_T) divided by the total number of randomized trials (K, K=5000.) **(D)** Example of randomly sampled Jaccard Null-distribution for the comparison of organotropic patterns for NIH 3T3 fibroblasts vs TC32 Ewing Sarcoma cell line. The experimental Jaccard value X is indicated in red, yielding a p-value of 0.031, i.e. the two patterns are deemed significantly different. See Fig. 4B. **(E)** Example of randomly sampled Jaccard Null-distribution for the comparison of organotropic patterns for sub-accumulation Sample A vs Sample B. The experimental Jaccard value X is indicated in red, yielding a p-value of 0.88, i.e. the two patterns are deemed statistically identical. See Fig. 4D. Note that the Null-distribution in (E) is narrower than in (D) because of the greater homogeneity of the cohort of fish making up the Null/joint population. This illustrates the need for specific sampling of Null-distributions with every comparison.

bioRxiv preprint doi: https://doi.org/10.1101/2025.05.28.656734; this version posted June 1, 2025. The copyright holder for this preprint (which was not certified by peer review) is the author/funder, who has granted bioRxiv a license to display the preprint in perpetuity. It is made available under aCC-BY 4.0 International license.

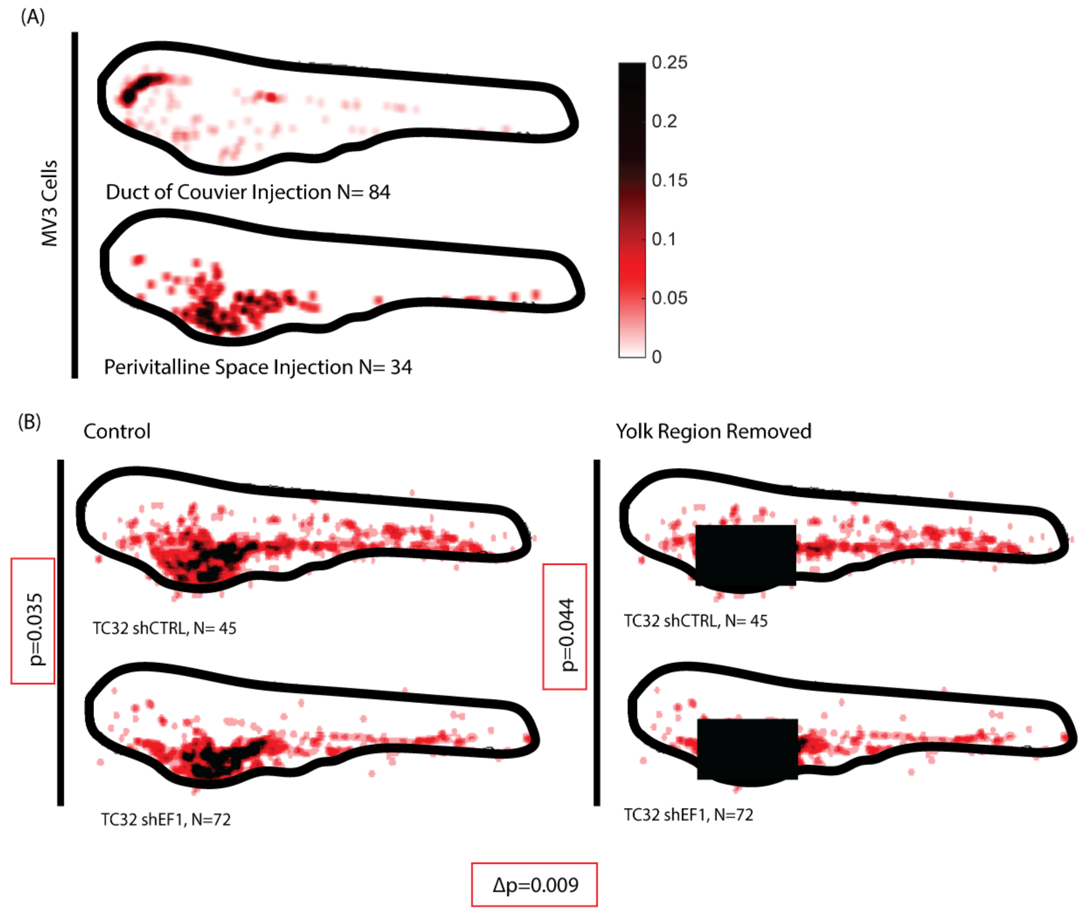

Figure S3 Effect of injection site choice and inclusion in the statistical comparison of Fish Metastatic Atlas pages. (A) Accumulators of MV3 xenotransplants into the Duct of Cuvier (top) and Perivitelline Space (bottom; this accumulator is similar to Fig. 4A). Box indicates P-value of a permutation test of the hypothesis that the two accumulators originate from the same sampling cohorts (see Methods). **(B)** Comparison of P-values of permutation tests of the hypothesis that TC32 expressing shCtrl or shEF1 (see Fig. Fig. 4F) generate the same sampling cohorts, once without (left) and once with the accumulator signal in the yolk mased. The difference in P-value between the two analyses is 0.009.
